# Differential Item Functioning Among English Language Learners on a Large-Scale Mathematics Assessment

**DOI:** 10.3389/fpsyg.2021.657335

**Published:** 2021-07-27

**Authors:** Ruixue Liu, Kelly D. Bradley

**Affiliations:** ^1^Department of Psychology, Henan Normal University, Xinxiang, China; ^2^Department of Educational Policy Studies and Evaluation, University of Kentucky, Lexington, KY, United States

**Keywords:** English language learners, achievement gap, mathematics assessment, differential Item functioning, PISA

## Abstract

The current study research showed the nature and potential sources of the gaps in mathematics achievement between English language learners (ELLs) and non-English language learners (non-ELLs). The nature of achievement gap was examined using three DIF methodologies: including Mantel-Haenszel procedure, Rasch model, and Hierarchical Generalized Linear Model (HGLM). These were conducted at the item level in contrast to total test level. Results revealed that the three DIF approaches identified 10 common items. These 10 items demonstrated in favor of non-ELLs. Findings from this study will help educational researchers, administrators, and policymakers understand the nature of the achievement gap in mathematics at item level so that United States can be competitive in middle school mathematics education. This study also suggested that item writers and test developers should construct assessments where language is equally accessible for ELL students.

## Introduction

According to the U.S. Department of Education, ELLs are defined as students “who are being served in appropriate programs of language assistance” (National Center for Education Statistics, 2016). In recent decades, ELLs are a rapidly growing student group in the United States. The percentage of public-school students in the United States identified as ELLs grew from 3.8 million (8.1%) in 2000 to 4.8 million (9.5%) in 2015. In Fall 2015, the percentage of public-school students who were ELLs ranged from 1.0% in West Virginia to 21.0% in California (National Center for Education Statistics, 2018).

In school classrooms across the United States, ELLs are learning English and the content material in their required academic subjects simultaneously. To this end, ELLs have been found to lag behind their non-ELL peers on large-scale, standardized assessments, largely due to the high language demand in content areas, such as mathematics, science, reading comprehension, writing, and social studies (Abedi and Lord, 2001; Abedi et al., 2001; Abedi, 2002; Johnson and Monroe, 2004; Ockey, 2007; Mahoney, 2008; Walker et al., 2008; Martiniello, 2009; Wolf and Leon, 2009).

### Purpose of Study

The current study aimed to explain the nature and potential sources of the gaps in mathematics achievement between ELLs and non-ELLs. The achievement gaps were examined using three differential item functioning (DIF) detection methods: Mantel-Haenszel (MH) procedure, Rasch model, and Hierarchical Generalized Linear Model (HGLM). Here, analyses were conducted at the item level instead of the traditional, total test result. Among the three methods, MH is the most widely used procedure to detect DIF in practice. The Rasch model (Rasch, 1960) allows for generalizability across samples and items, and it can identify poorly functioning items as well as unexpected responses. HGLM takes into account the nested structure of data where items are nested within students and students are nested within schools. At the student level, sources of DIF were investigated through the students' variations in mathematics self-efficacy, language proficiency, and socioeconomic status (SES). At the school level, school type and school educational resource were investigated as potential sources of DIF after controlling for the student variables.

The language of mathematics has been viewed as a unified system of meaning-making that incorporates multiple semiotics (Martiniello, 2009). The need to allocate cognitive resources to comprehend a problem presented in a non-primary language would reduce the resources available for the problem-solving process and result in increasing the probability of errors (Barbu, 2010). Therefore, it is necessary to investigate the assessment validity of mathematics for ELLs.

The Programme for International Student Assessment (PISA) is a large-scale assessment. The resulting data set allows researchers to investigate academic achievement and group membership from a variety of different viewpoints (Organisation for Economic Co-Operation Development, 2014). In this study, the U.S. sample of PISA 2012 was used. Seventy-six dichotomously coded items from PISA 2012 mathematics assessment were included to detect DIF effects.

### Research Questions

Specifically, the present study will mainly address three research questions.

1. Do items from PISA 2012 mathematics assessment exhibit DIF between ELLs and non-ELLs for the U.S. sample?
2. If DIF is detected, can English language proficiency and other student characteristics (e.g., student SES, mathematics self-efficacy) explain DIF? That is, after controlling for these three student variables, whether DIF between ELLs and non-ELLs changes was examined.
3. If DIF is detected, can school type and school educational resources contribute to DIF?

The first research question aims to explore whether the PISA 2012 mathematics items are measuring in essentially the same way for ELLs and non-ELLs. The second and third research questions, incorporating a multilevel item analysis method, aim to identify the problem from multiple perspectives. Findings from this study can help educational researchers, administrators, and policymakers understand the nature of the achievement gap at item level instead of the total test level so the United States can be competitive in middle school mathematics education. This study also suggested that item writers and test developers should construct assessments where language is equally accessible for ELL students. The significance of this study lies in the empirical investigation of the gap between ELLs and non-ELLs in mathematics achievement at an item level and from the perspectives of both students and schools.

## Literature Review

### Assessment Validity for ELLs

Validity, as one of the most important attributes of an assessment, refers to how well the assessment tool measures the underlying outcome of interest. Validity is not a property of the tool itself, but rather of the interpretation or specific purpose of the assessment tool with particular settings and learners (American Educational Research Association et al., 2014). For ELLs, as well as for all populations, it is critical to consider whether the test scores can reflect the skill or proficiency that an assessment is intended to measure. Although students may have different English proficiency, the meaning of their scores on content assessments should be comparable (Educational Testing Service, 2009).

According to the Standard for Educational and Psychological Testing, “*the linguistic or reading demands of the test should be kept to the minimum necessary for the validity of assessment for the intended construct*” (American Educational Research Association et al., 2014, p. 82). Since ELL test takers have not yet acquired sufficient mastery of the English language, high language demand is still evident on mathematics assessment (Loughran, 2014). In this case, to a certain degree, the test unintentionally measures language proficiency. The lack of English proficiency used to comprehend mathematics assessment items results in an increased cognitive load and contributes to measurement error of ELL students' mathematics content knowledge (Educational Testing Service, 2009).

### Differential Item Functioning

According to Holland and Wainer (1993), DIF analysis is a statistical technique to identify whether items on an assessment are of equal difficulty for examinees of different groups. DIF is present if an item on a test functions differently for different groups of interest (e.g., ELLs vs. non-ELLs), given the ability level. In the DIF analysis, examinees are matched based on their underlying ability (e.g., total score of an assessment), and differences in item performance between groups of examinees at the same level of ability are then determined.

### DIF Detection Methods

There are numerous statistical methods for detecting DIF. The following methods are frequently used: Mantel-Haenszel procedure (Holland and Thayer, 1988), SIBTEST (Shealy and Stout, 1993), Item Response Theory methods (Camille and Shepard, 1994), logistic regression (Swaminathan and Rogers, 1990), and multilevel DIF analysis (Kamata, 2001). In this section, three methods applied in the current study were discussed in particular.

#### Mantel-Haenszel Procedure

The MH statistic was applied by Holland and Thayer (1988) in determining DIF. The MH DIF procedure compares dichotomous item performance between two groups after matching respondents on overall scores. Respondents in the focal and reference groups were matched on total test scores by dividing respondents in both groups into defined strata on those scores. The total scores were generated by summing item scores across all items. Estimates of the odds ratio for a given item can be calculated based on a 2 × 2 × K contingency table with k representing the *k-*th group (*k* = 1,2,…K). Table 1 shows the 2 × 2 contingency table for the *k*-th group of an item. The A_k_, B_k_, C_k_, and D_k_ denote the numbers of respondents in the cells. T_k_ represents the number of respondents in the k-the stratum.

**Table 1:** 2 × 2 × K contingency table.

	**Scores on the Studied items**		
	**1 (Right)**	**0 (Wrong)**	**Total**
Reference group	A_k_	B_k_	N_rk_
Focal group	C_k_	D_k_	N_fk_
Total	M_1k_	M_0k_	T_k_

The cells A_k_ and C_k_ represent the total number of respondents who answered the item correctly in the reference and focal groups, respectively, within the matched subgroup k. B_k_ and D_k_ denote the total number of respondents who answered the item incorrectly in the reference and focal groups, respectively, within subgroup k. N_rk_ and N_fk_ are the total number of respondents in the reference and focal groups, respectively, within k-th group. M_1k_ and M_0k_ denote the number of respondents who answered the item correct and incorrect, respectively, within k-th group.

The MH chi-square statistic is used for testing the null hypothesis of whether the population odds of getting an item correct is the same in the reference and focal groups. The statistic is given by

(1) $$\begin{array}{l} \text{MH CHISQ}=\frac{{\left(|\sum_{k=0}^{n}{\text{A}}_{k}-\sum_{k=0}^{n}\text{E}\left({\text{A}}_{k}\right)|-\frac{1}{2}\right)}^{2}}{\sum_{k=0}^{n}\text{Var}\left({\text{A}}_{k}\right)} \end{array}$$

In this equation, E(A_k_) and Var(A_k_) follow:

(2) $$\begin{array}{l} \text{E}\left({\text{A}}_{\text{k}}\right)=\frac{N_{rk}\;*M_{1k}}{T_{k}} \end{array}$$

(3) $$\begin{array}{l} \text{Var}\left({\text{A}}_{\text{k}}\right)=\frac{N_{rk}\;*N_{fk}*M_{1k}*M_{0k}}{\left[\;T_{K^{2}}\;\left(T_{K}-1\right)\right]} \end{array}$$

The common odds-ratios formula is:

(4) $$\begin{array}{l} \text{O}{\text{R}}_{\text{MH}}=\overset{n}{\underset{k=0}{\sum }}\left[\frac{{\text{A}}_{k}*{\text{D}}_{k}}{{\text{T}}_{k}}\right]/\overset{n}{\underset{k=0}{\sum }}\left[\frac{B_{k}*C_{k}}{T_{k}}\right] \end{array}$$

The scale for OR_MH_ is from 0 to ∞, with OR_MH_ = 1 denoting the case of no DIF. For convenience, OR_MH_ is converted into a symmetrical scale Δ OR_MH_ given as

(5) $$\begin{array}{l} \Delta {\text{OR}}_{\text{MH}}=-2.35ln\left({\text{OR}}_{\text{MH}}\right) \end{array}$$

Δ OR_MH_ is applied as a measure of the DIF effect size. Educational Testing Service classified the DIF effect size as follows (Dorans and Holland, 1993) to aid in interpretation in applications: Class A denotes negligible magnitudes of DIF, when |Δ OR_MH_| < 1.00; Class B denotes moderate magnitudes of DIF, when 1.00 ≤| Δ OR_MH_| < 1.50, and Class C denotes large magnitudes of DIF when |Δ OR_MH_| ≥ 1.50.

The validity of the MH DIF detection method has been established by numerous studies (Qian, 2011). It is the most widely used procedure to detect DIF in practice since it is not only easy to understand and compute, it can provide both a significance test and estimate of the magnitude of DIF as well (Millsap, 2011). The major criticism of the MH procedure is the adequacy of using the total score as a substitute for the latent trait (Millsap, 2011). Besides, the requirement for sample sizes is a technical challenge to detecting items with DIF since DIF statistics become less stable as sample sizes decrease.

#### Rasch Model

Rasch model (Rasch, 1960) can produce a comprehensive and informative picture of the construct under measurement as well as the respondents on that measure. Since PISA 2012 employs the Rasch model to estimate student ability, item difficulty, and create the overall PISA literacy scale (Organisation for Economic Co-Operation Development, 2013), Rasch model was selected to detect DIF in the current study. Rasch model follows mathematically from the requirement of invariance of comparisons among persons and items (Andrich and Luo, 2003). Rasch model follows the following form:

(6) $$\begin{array}{l} P_{ij}\left(Y_{ij}=1|\theta_{j},b_{i}\right)=\frac{e^{\left(\theta_{j}\;-\;b_{i}\;\right)}}{\left[1+\;e^{\left(\theta_{j}\;-\;b_{i}\right)}\right]} \end{array}$$

where p_ij_ is the probability of person j answering correctly to item i. θ_j_ is the person trait, or ability. b_i_ is the item parameter indicating difficulty of the item.

Rasch model provides a theoretically useful way to detect DIF which can be modeled using estimated item parameters and ability. A popular approach for detecting the DIF using the Rasch model is the Rasch separate calibration *t*-test method. This method is based on the differences between two separate calibrations of the same item from the subpopulation of interest, holding other item and person calibrations constant to ensure scale stability (Wright and Stone, 1979; Peabody and Wind, 2019). In Winsteps (Linacre, 2017), DIF detection using the Rasch model is conducted by a subtraction of the item location parameters (item difficulties) for two groups, d_1_ and d_2_. They are converted to standard normal variates using a pooled standard error. DIF is detected if the difference of item location parameters is statistically different.

(7) $$\begin{array}{l} t=\frac{d_{1}-d_{2}}{\sqrt{var\;\left(d_{1}\right)+var\;\left(d_{2}\right)}} \end{array}$$

#### Hierarchical Generalized Linear Model

In behavioral and social sciences, data commonly have a nested structure. For example, repeated observations are nested within persons (e.g., responses nested within examinees), and persons are nested within organizational units such as classrooms, schools, and communities, and so on (Raudenbush and Bryk, 2002). Kamata (2001) proposed to use HGLM to detect DIF effects. Rasch model has been shown to be a special case of HGLM (Kamata, 2001; Raudenbush et al., 2003). In the three-level HGLM, the first level of the model is the item level, the second level the student level, and the third level is the school level. The three-level models have a nested structure where items are nested within students, and students are nested within schools.

Kamata (2001) mentioned that the three-level HGLM would be useful when the variation of the effect of a student characteristic variable across groups and the identification of a group-characteristic variable that explains such variation are of interest. There are several advantages of using HGLM to detect DIF in large-scale assessments. First, since the dependency of the data due to the nested data structure can be considered, DIF and item difficulty parameters can be modeled randomly across schools. Then the student and school variables can be examined simultaneously as potential sources of DIF. Second, additional student-level variables can be added as covariates to reduce student variations when identifying DIF. Third, various sources of DIF unique to each DIF item can be modeled simultaneously. Fourth, DIF detection using HGLM does not require two separate groups (focus and reference groups). This is especially beneficial if the source of the hypothesized DIF is a continuous variable (Qian, 2011).

## Method

### Data Source

The primary database used in this research is constructed from the PISA 2012. PISA is the most comprehensive and rigorous international assessment on 15-year-old students' performance in mathematics, reading, and science. According to the National Center for Education Statistics (2016), students in the PISA 2012 U.S. sample were born between July 1, 1996, and June 30, 1997. Finally, the PISA 2012 U.S. sample contains 4,978 students from 162 schools.

The targeted population for the focal group in this study is ELL students. Groups were identified using information collected from Student Questionnaire that was administered with the test. Basically, these two groups were designed to differ only in their relationships with English (as a first or second language). Home Language (ST25Q01) was used to form the groups. Home Language has the following binary categories: (1) language at home is the same as the language of the test and (2) language at home is another language. Students failing to answer this question were excluded from the current study. Finally, 670 students were identified as ELL students while 4,196 students were identified as non-ELL students.

## Measures

### Mathematics Items

In this study, 76 dichotomously coded items from PISA 2012 mathematics assessment were analyzed (See Appendix A). These items are either selected response multiple-choice or closed-constructed response. These items were scored as correct or incorrect and coded dichotomously with 1 and 0.

### Student-Level Measures

The second research question aimed to investigate whether mathematics self-efficacy, English language proficiency, and student SES can explain DIF. These three variables were selected since they were found to be significant predictors to influence mathematics performance for ELLs (Aikens and Barbarin, 2008; Guglielmi, 2012). In addition, these variables served as control variables to reduce student variations. The measures of mathematics self-efficacy, language proficiency, and student SES were introduced in this section.

#### Mathematics Self-Efficacy

According to Bandura (1997), self-efficacy or perceived ability refers to the confidence an individual has in their ability to successfully perform a specific task. Previous studies indicated that mathematics self-efficacy and mathematics achievement were positively related. Students with high mathematics self-efficacy are associated with high mathematics achievement (e.g., Ayotola and Adedeji, 2009).

In PISA 2012, eight items were used to measure mathematics self-efficacy. These items ask students how confident they feel about having to do eight tasks (See Appendix B). Mathematics self-efficacy was measured in a four-point Likert-type scale (1 = Very confident; 2 = Confident; 3 = Not very confident; and 4 = Not at all confident). These items were scaled using IRT scaling methodology (Organisation for Economic Co-Operation Development, 2013).

#### Language Proficiency

When using HGLM to detect DIF between ELL and non-ELL students, language proficiency was used as one of the covariates. However, this information is not available in PISA 2012. Reading literacy, a proxy for language proficiency was used to represent language proficiency since understanding written text is the first form of language proficiency relevant to cognitive functions (Chen, 2010). PISA 2012 assessed reading literacy based on students' performance on three broad aspect categories including the ability to access and retrieve, integrate and interpret, and reflect and evaluate. These aspects were evaluated on printed and electronic texts which were defined as description, narration, exposition, argumentation, instruction, and transaction. In addition, IRT was used to estimate average scores for reading literacy (Organisation for Economic Co-Operation Development, 2014).

#### Socioeconomic Status

This study utilized the PISA index of economic, social, and cultural status (ESCS) to represent student SES. Variables comprising ESCS included home possessions (HOMEPOS), the number of books at home (HISEI), and the highest parental education expressed as years of schooling (PARED). The ESCS scores were obtained as component scores for the first principal component with zero being the score of an average OECD student and one being the standard deviation across equally weighted OECD countries. ESCS scores were calculated using the following formula:

(8) $$\begin{array}{l} \text{ESCS}=\frac{\beta 1^{*}\text{HOMEPOS }+\;\beta 2^{*}\text{HISEI}+\beta 3^{*}\text{PARED}}{\varepsilon } \end{array}$$

where β_1_, β_2_, and β_3_ are the OECD factor loadings and ε is the eigenvalue of the first principal component (Organisation for Economic Co-Operation Development, 2014).

### School-Level Measures

The third research question aimed to investigate whether school type and school educational resources can contribute to DIF after controlling for student-level variables. These two variables were selected since they were found to be significant predictors to influence mathematics performance for ELLs (Freeman and Crawford, 2008; Han and Bridglall, 2009).

#### School Type

In PISA 2012, schools were categorized into public and private according to whether a private entity or a public agency has the ultimate power to make decisions concerning its affairs. The dummy variable of school type (SCHLTYPE) was created (0 = public, 1 = private).

#### School Educational Resources

The PISA 2012 school questionnaire contained 13 items about school educational resources, measuring principals' perceptions of potential factors hindering instruction at schools (e.g., a lack of qualified science teachers; shortage or inadequacy of science laboratory equipment; shortage or inadequacy of computer software for instruction; shortage or inadequacy of audio-visual resources). A four-point Likert-type scale was used (1 = Not at all, 2 = Very little, 3 = To some extent, 4 = A lot) (Organisation for Economic Co-Operation Development, 2014). The detailed items can be found in Appendix C. Responses to the 13 items measuring school educational resources were summed up and rescaled to a Z score to form the predictor of school educational resources.

## Statistical Analyses

### Mantel-Haenszel Procedure

The PROC FREQ under the software of SAS 9.4 was used to conduct the MH procedure (Zhang, 2015). The total scores generated by summing item scores across all items were used to match students. Students' proficiency levels were controlled by stratifying students into five stratums based on the total scores. The DIF procedures in SAS 9.4 can provide key statistics including MH chi-square, common log-odds ratio, and estimated standard error. The MH chi-square statistic is distributed as chi-square with one degree of freedom. An alpha level of 0.01 was used for the MH procedure flag DIF items. The MH odds ratio is asymptotically normally distributed. ETS guidelines were used to classify items displaying DIF effects.

### Rasch Model

The Rasch model was completed in the Winsteps measurement software, Version 3.9.1. Item difficulty measures (*b*_*i*_ in Equation 6) for both groups were calculated to examine whether the property of invariance was met. Winsteps outputs for DIF are equivalent to construct a “ruler” based on the persons, and measuring the items on it, first for the one person-group, then for the other person-group. In the output, the DIF contrast is the effect size of DIF and is a log-odds estimate. Specifically, the DIF contrast refers to the difference of item difficulty measures between ELLs and non-ELLs. A negative DIF contrast indicates that the item is more difficult for the ELLs.

### Hierarchical Generalized Linear Model

The current study also used HGLM on DIF detection based on Kamata (2001) and Binici's (2007) studies. This study discussed that HGLM is equivalent to the Rasch model and showed how the two-level HGLM can be extended to a three-level latent regression model. Specifically, three models were created to answer the three research questions. Model 1 (DIF Identification Model) examined each item for DIF between ELLs and non-ELLs. Model 2 (DIF Estimation Model Controlling for Student-Level Variables) further examined whether student-level variables (mathematics self-efficacy, language proficiency, and SES) can explain DIF. Finally, Model 3 (Random Effects DIF Model) included student-level variables and school-level (school type and school educational resource) variables to explain DIF.

Model 1, including Level-1 and Level-2 models, was applied to the 76 items to detect DIF effects. Level-1 model is specified as given by Equation (9) where the log odds of the probability of answering each item correctly vs. incorrectly is a linear function of person ability and item difficulty.

(9) $$\begin{array}{l} Log\left(\frac{p_{ij}}{1-p_{ij}}\right)=\beta_{0j}+\overset{76}{\underset{q=1}{\sum }}\left(\beta_{qj}*X_{qij}\right) \end{array}$$

*X*_*qij*_ is the *q*-th (q = 1, 2, …, 76) dummy coded variable that indicates the item *i* for student *j*. Its value is 1 when *q* = *i* and 0 when *q* ≠ *i*. β_0*j*_ is the effect of the reference item and β_*qj*_ is the difference between the *q*-th item and the reference item. The probability of student *j* getting an item *i* correct is noted as *p*_*ij*_.

Level-2 model was then created by adding the group membership (ELL status) and modeling regression coefficients, β*qj* (*q* = 1, 2,…, 76) in Equation (9) as given by Equation (10).

(10) $$\begin{array}{l} \beta_{0j}=\;\tau_{00}+u_{0j}\\ \beta_{1j}=\;\tau_{10}+\tau_{11}*ELL\\ \ldots \\ \beta_{76j}=\;\tau_{760}+\tau_{761}*ELL \end{array}$$

In Equation (10), coefficients τ_11_ to τ_761_ are the DIF coefficients associated with items 1 through 76. ELL students were coded as 1 and non-ELL students were coded as 0. A significant DIF coefficient indicates the existence of DIF for the item under investigation. The exponential term of the DIF coefficient is the odds of answering the corresponding item correctly by the reference vs. the focal group.

Model 2 further examined whether DIF effects decreases or disappears after controlling for student-level variables. As shown in Equation (11), Model 2 was created by adding student-level variables (language proficiency, SES, and mathematics self-efficacy) to Level-2 of Model 1. Level-1 of Model 2 is the same with Level-1 of Model 1 as shown as Equation (10). Level-2 of Model 2 was specified as follows.

(11) $$\begin{array}{l} \beta_{0j}=\;\tau_{00}+u_{0j}\\ \beta_{1j}=\;\tau_{10}+\tau_{11}*ELL\;+\;\tau_{12}*Language\;+\;\tau_{13}*SES+\tau_{14}*SE\\ \beta_{2j}=\;\tau_{20}+\tau_{21}*ELL\;+\;\tau_{22}*Language\;+\;\tau_{23}*SES+\tau_{24}*SE\\ \ldots \\ \beta_{76j}=\;\tau_{760}+\tau_{761}*ELL\;+\;\tau_{762}*Language\;+\;\tau_{763}*SES\;+\tau_{764}*SE \end{array}$$

In Equation (11), coefficients τ_11_ to τ_761_ are the estimates of DIF after controlling for student-level variables. The exponential term of the DIF coefficients is the odds of answering the corresponding item correctly by the reference vs. the focus group. Coefficients τ_12_ to τ_762_ are the log odds of answering the corresponding item correctly with one unit of standard deviation (SD) increase in language proficiency. Similarly, τ_13_ to τ_763_ and τ_14_ to τ_764_ indicate the log odds of answering the corresponding item correctly with one unit of SD increase in SES and mathematics self-efficacy.

Model 3 examined whether school type and school educational resource contribute to the DIF. Specifically, Model 3 is a three-level DIF identification model including student-level and school-level variables. It investigated whether school type and school educational resources were significant predictors of DIF variations among 162 schools between ELL and non-ELL students.

DIF items that were detected by Model 1 were included in the analysis of Model 3. The Level-1, Level-2, and Level-3 of Model 3 were specified as shown in Equations (12, 13, and 14).

Level-1:

(12) $$\begin{array}{l} \mathrm{Log}\left(\frac{p_{ijk}}{1-p_{ijk}}\right)=\beta_{0j}+\overset{n}{\underset{q=1}{\sum }}\left(\beta_{qjk}*X_{qijk}\right) \end{array}$$

Level-2:

(13) $$\begin{array}{l} \beta_{0jk}=\;\tau_{00k}+u_{0jk}\\ \beta_{1jk}=\;\tau_{10k}+\tau_{11k}*ELL\;+\;\tau_{12k}*Language\;+\;\tau_{13k}*SES+\;\tau_{14k}*SE\\ \ldots \\ \beta_{njk}=\;\tau_{n0k}+\tau_{n1k}*ELL\;+\;\tau_{n1k}*Language\;+\;\tau_{n1k}*SES\;+\;\tau_{n1k}*SE \end{array}$$

Level-3:

(14) $$\begin{array}{l} \tau_{00k}=\;\pi_{000}+\varepsilon_{00k}\\ \tau_{10k}=\;\pi_{100}\\ \tau_{11k}=\;\pi_{110}+\pi_{111}*Schooltype+\;\pi_{112}*Resources\\ \tau_{12k}=\;\pi_{120}\\ \tau_{13k}=\;\pi_{130}\\ \tau_{14k}=\;\pi_{140}\\ \tau_{20k}=\;\pi_{200}\\ \tau_{21k}=\;\pi_{210}+\pi_{211}*Schooltype+\;\pi_{212}*Resources\\ \ldots \\ \tau_{100k}=\;\pi_{1000}\\ \tau_{nk}=\;\pi_{n10}+\pi_{n11}*Schooltype+\;\pi_{n12}*Resources\\ \ldots \end{array}$$

The subscripts n and k indicate n-th DIF item and k-th school, respectively, at Level-3. At Level-2, coefficients of τ_11*k*_ to τ_*n*1*k*_ are random DIF coefficients that vary from school to school. At Level-3, coefficients π_111_ to π_*n*11_indicate how much DIF increases when a school is from public to private, and coefficients π_112_ to π_*n*12_ denote how much DIF increases when school educational resources increase by one unit of SD. π_120_ to π_*n*20_ are the fixed regression coefficients for language proficiency. π_130_ to π_*n*30_ are the fixed regression coefficients for SES. π_130_ to π_*n*30_ are the fixed regression coefficients for mathematics self-efficacy.

The application of HGLM to detect DIF was conducted with PROC GLIMMIX under the software SAS 9.4. This procedure can fit models to outcome variables that generate a linear model with explanatory variables that account for variations at each level, utilizing variables specified at each level. PROC GLIMMIX can not only estimate model coefficients at each level, but it also predicts the random effects associated with each sampling unit at every level.

## Results

### Mantel-Haenszel Procedure

The MH procedure was the first approach in this study to examine DIF effects between ELL and non-ELL students. Table 2 shows the results of DIF effects using the MH procedure. Among the 76 items, 59 items with negligible DIF were categorized into Class A. Seven items with moderate values of ΔOR_MH_ were categorized into Class B. Ten items with large values of ΔOR_MH_ were categorized into Class C. In Class B, all seven items were in favor of non-ELL students. For example, Item 8 with the odds ratio of 0.55 indicated that ELL students are 45% less likely to answer this item correctly. In Class C, all ten items were in favor of non-ELL students. For example, Item 16 with the odds ratio of 0.50 indicated that ELL students are 50% less likely to answer this item correctly.

**Table 2:** Summary results from the MH procedure to identify DIF effects.

**Item number**	**MH chi-square**	**Odds-ratio**	**DIF effect size**	**Class**
8. PM192Q01T	12.76^**^	0.55	1.42	B
16. PM420Q01T	21.23^**^	0.50	1.63	C
40. PM909Q01	14.84^**^	0.39	2.24	C
41. PM909Q02	20.02^**^	0.51	1.59	C
42. PM909Q03	31.67^**^	0.34	2.57	C
43. PM915Q01	13.10^**^	0.55	1.42	B
46. PM918Q02	23.31^**^	0.47	1.80	C
47. PM918Q05	7.78^**^	0.64	1.05	B
49. PM919Q02	10.10^**^	0.62	1.12	B
56. PM949Q01T	14.22^**^	0.56	1.35	B
57. PM949Q02T	14.00^**^	0.50	1.61	C
61. PM954Q02	16.74^**^	0.51	1.59	C
63. PM955Q01	16.90^**^	0.51	1.57	C
64. PM955Q02	11.75^**^	0.48	1.73	C
68. PM982Q04	9.52^**^	0.60	1.20	B
73. PM995Q02	5.11^**^	0.18	4.05	C

** *p ≤ 0.01*.

### Rasch Model

DIF can be examined within the Rasch model by comparing item difficulties between groups. Table 3 reports the difficulty estimates for both groups and their difficulty contrast. The t statistics were calculated using Equation (7). If the difficulty measures are significantly different between ELL and non-ELL students for the same item, this item was considered to have a DIF issue. A positive difficulty contrast indicates the item is more difficult for non-ELL students, and a negative difficulty contrast implies the item is more difficult for ELL students. According to Table 2, 14 items have been found to have DIF effects. Among the 14 items, nine items were more difficult for ELL students and five items were more difficult for non-ELL students. According to de Ayala (2009), items with difficulty contrast above 0.30 are considered as being noticeable. Thus these 14 items were found to display practically significant DIF effects.

**Table 3:** Summary of results from Rasch model to identify DIF effects.

**Item number**	**Difficulty measures**		**Difficulty contrast**	***t***	**df**
	**Non-ELLs**	**ELLs**			
8. PM192Q01T	0.25	0.66	−0.40^*^	−1.98	343
16. PM420Q01T	−0.88	−0.48	−0.40^*^	−2.28	394
27. PM564Q01	0.09	−0.48	0.48^**^	2.61	360
34. PM828Q02	−0.79	−1.37	0.58^**^	3.32	389
40. PM909Q01	−3.62	−3.08	−0.54^*^	−2.23	484
42. PM909Q03	0.82	1.56	−0.74^**^	−3.08	338
43. PM915Q01	−0.06	0.42	−0.48^*^	−2.45	326
46. PM918Q02	−2.04	−1.50	−0.53^**^	−2.98	464
49. PM919Q02	0.19	0.49	−0.30^*^	−1.67	416
51. PM923Q03	0.14	−0.41	0.54^**^	3.16	429
55. PM943Q02	4.69	3.66	1.03^**^	2.44	495
61. PM954Q02	0.65	1.21	−0.56^*^	−2.76	398
68. PM982Q04	0.01	0.36	−0.35^*^	−1.80	329
76. PM998Q04T	0.81	0.03	0.78^**^	4.19	385

* *p ≤ 0.05*;

** *p ≤ 0.01*.

### Hierarchical Generalized Linear Model

#### Model 1

Table 4 summarizes the results of items with DIF effects using HGLM. Estimates are the DIF coefficients in Model 1. Estimates were exponentiated to obtain the DIF odds ratios. Then odds ratios were transformed to DIF effect size (Δ OR_MH_). Similar to the MH procedure, effect sizes were categorized into three classes. Among the 76 items, 66 items with negligible DIF were categorized into Class A. Five items with moderate values of ΔOR_MH_ were categorized into Class B. Six items with large values of ΔOR_MH_ were categorized into Class C. In Classes B and C, all the items were in favor of non-ELLs. For example, Item 8 with the DIF odds ratio of 0.62 indicated that ELL students are 38% less likely to answer this item correctly.

**Table 4:** Summary of HGLM Model 1.

**Item number**	**Estimates**	**Odds ratio**	**DIF effect size**	**Class**
8. PM192Q01T	−0.47^**^	0.62	1.12	B
16. PM420Q01T	−0.52^**^	0.60	1.21	B
40. PM909Q01	−0.70^**^	0.50	1.65	C
42. PM909Q03	−0.87^**^	0.42	2.04	C
43. PM915Q01	−0.56^**^	0.57	1.32	B
46. PM918Q02	−0.64^**^	0.53	1.51	C
49. PM919Q02	−0.40^**^	0.65	1.01	B
61. PM954Q02	−0.64^**^	0.53	1.50	C
68. PM982Q04	−0.45^**^	0.64	1.05	B
73. PM995Q02	−1.60^**^	0.20	3.77	C

** *p ≤ 0.01*.

#### Model 2

In Model 2, student-level variables including mathematics self-efficacy, language proficiency, and SES were included to identify whether they are the potential sources of DIF between ELLs and non-ELLs. If the number of items showing DIF effects and their effect sizes decrease after controlling for the student-level variables, these three variables are the potential sources of DIF at the student-level.

Table 5 displays the results from Model 2 to identify DIF effects controlling for student-level variables. Estimates are the DIF coefficients in Model 2. Odds ratios of student-level variables are the exponential terms for the regression coefficients of mathematics self-efficacy, language proficiency, and SES, which indicate the odds of getting each item correct associated one SD increase in those three variables.

**Table 5:** Summary of results from HGLM Model 2.

**Item number**	**Estimates**	**DIF Odds ratio**	**DIF effect size**	**Odds ratios of student-level variables**		
				**Mathematics self-efficacy**	**Language proficiency**	**SES**
8. PM192Q01T	−0.46^*^	0.63	1.08	1.58^**^	1.01^**^	0.86
16. PM420Q01T	−0.36	0.70	0.85	1.06	1.01^**^	1.09
40. PM909Q01	−0.67^*^	0.51	1.58	1.02	1.01^**^	0.92
42. PM909Q03	−0.57^*^	0.56	1.34	1.03^**^	1.01^**^	1.21
43. PM915Q01	−0.46^*^	0.63	1.09	1.02^**^	1.01^**^	1.08^*^
46. PM918Q02	−0.59^**^	0.55	1.39	1.00	1.01^**^	0.99
49. PM919Q02	−0.42^*^	0.66	0.99	1.02	1.01^**^	0.91
61. PM954Q02	−0.50^*^	0.61	1.18	1.02^**^	1.01^**^	1.18^*^
68. PM982Q04	−0.28	0.75	0.66	1.18^**^	1.01^**^	1.14
73. PM995Q02	−1.46	0.23	3.43	1.18^**^	1.02^**^	1.11

* *p ≤ 0.05*;

** *p ≤ 0.01*.

Mathematics self-efficacy was a significant predictor on six of ten DIF items. For example, Item 8 with the odds ratio of 1.58 indicated that students with one SD increase of mathematics self-efficacy were 1.58 times more likely to answer this item correctly. Language proficiency was a significant predictor for all 10 items even its effect was minimal. For example, Item 73 with the odds ratio of 1.02 indicated that students with one SD increase of language proficiency were 1.02 times more likely to answer this item correctly. SES was a significant predictor on four of ten DIF items. For example, Item 61 with the odds ratio of 1.18 indicated that students with one SD increase of SES were 1.18 times more likely to answer this item correctly. After controlling for student-level variables, seven items still displayed DIF effects while the remaining three items no longer showed DIF effects. Besides, all the DIF effect sizes decreased after controlling for student-level variables.

#### Model 3

The three-level model was implemented for the 10 items displaying DIF effects. In this model, DIF effects were modeled to vary across 157 schools after controlling for the three student-level variables. Items with significant DIF variations across the schools were identified.

Table 6 displays the results from Model 3 to identify DIF effects controlling for student and school-level variables. Only odds ratios of school-level variables were displayed. Three out of 10 items were found to show significant DIF effects as both student and school-level variables were controlled (Item 8, 40, and 46). Nevertheless, school educational resources were not a significant predictor for these 10 items. School type was found to be a significant predictor for Item 40 and 46. For Item 40, students in private schools are 1.17 times more likely to answer this item correctly. For Item 46, students in private schools are 1.64 times more likely to answer this item correctly.

**Table 6:** Summary of results from HGLM Model 3.

**Item number**	**Estimates**	**DIF odds ratio**	**DIF effect size**	**Odds ratios of school-level variables**	
				**School type**	**School educational resource**
8. PM192Q01T	−0.66^*^	0.52	1.56	1.06	0.98
16. PM420Q01T	−0.45	0.64	1.06	0.70	0.94
40. PM909Q01	−0.60^*^	0.44	1.41	1.17^*^	0.98
42. PM909Q03	−0.56	0.57	1.31	0.76	1.07
43. PM915Q01	−0.46	0.63	1.09	1.08	0.97
46. PM918Q02	−0.78^*^	0.46	1.84	1.64^*^	0.94
49. PM919Q02	−0.17	0.84	0.40	0.97	0.97
61. PM954Q02	−0.25	0.78	0.59	0.82	1.00
68. PM982Q04	−0.51	0.60	1.19	1.06	0.95
73. PM995Q02	−1.37	0.25	3.22	1.01	0.96

* *p ≤ 0.05*.

### Consistency of Three DIF Detection Methods

Table 7 summarizes the DIF items identified by MH procedure, Rasch model, and HGLM. Seventeen items were identified with DIF effects in one of those three methods. Among those 17 items, eight items (Items 8, 16, 40, 43, 46, 49, 61, and 68) were identified with DIF effects by all three methods. Besides, the MH approach discovered six items with DIF effects that were not identified by the other two methods. Rasch model found five items with DIF effects that were not identified by the other two methods.

**Table 7:** Summary of DIF items identified by three methods.

**Item number**	**Mantel-Haenszel procedure**	**Rasch model**	**HGLM**
8. PM192Q01T	Yes	Yes	Yes
16. PM420Q01T	Yes	Yes	Yes
27. PM564Q01		Yes	
34. PM828Q02		Yes	
40. PM909Q01	Yes	Yes	Yes
41. PM909Q02	Yes		
42. PM909Q03	Yes	Yes	Yes
43. PM915Q01	Yes	Yes	Yes
46. PM918Q02	Yes	Yes	Yes
47. PM918Q05	Yes		
49. PM919Q02	Yes	Yes	Yes
51. PM923Q03		Yes	
55. PM943Q02		Yes	
56. PM949Q01T	Yes		
57. PM949Q02T	Yes		
61. PM954Q02	Yes	Yes	Yes
63. PM955Q01	Yes		
64. PM955Q02	Yes		
68. PM982Q04	Yes	Yes	Yes
73. PM995Q02	Yes		Yes
76. PM998Q04T		Yes	

## Discussions

### Summary of Findings

The first research question in this study asked whether 76 dichotomous items from PISA 2012 mathematics assessment exhibit DIF between ELLs and non-ELLs. In total, 21 items were identified with DIF effects by any of three methods. Sixteen items were found to be more difficult for ELLs while five items were more difficult for non-ELLs. Five items that were in favor of ELLs were identified by the Rasch model. Besides, eight items (Items 8, 16, 40, 43, 46, 49, 61, and 68) were identified with DIF effects by all three methods. These eight items were found to be more difficult for ELLs. the MH approach discovered six items with DIF effects which were not identified by the other two methods. The Rasch model found five items with DIF effects that were not identified by the other two methods.

Among the 10 DIF items that were identified by HGLM, seven items still displayed DIF effects after controlling for student-level variables. The rest of three items no longer showed DIF effects. These results suggest that mathematics self-efficacy, language proficiency, and SES are potential sources of DIF between ELLs and non-ELLs. Moreover, mathematics self-efficacy was a significant predictor on six of 10 DIF items. Language proficiency was a significant predictor for all 10 items even its effect was minimal. SES was a significant predictor on four of 10 DIF items. In addition, three items still displayed DIF effects after controlling for both student and school-level variables. The rest of seven items no longer displayed DIF effects. School type is a significant predictor for two items, while school educational resources were not a significant predictor for these 10 items.

Three DIF detection methods consistently identified eight items which were in favor of non-ELLs. This finding demonstrated the impact of English language proficiency on mathematics assessment, which aligned with some early studies. For instance, Abedi (2002) utilized existing data from several locations across the U.S. to examine the impact of students' language background on mathematics performance. The analyses mainly focused on the comparison between the level of performance of ELL and non-ELL students. The results discovered that ELLs generally perform lower than non-ELL students in mathematics. Similarly, Beal et al. (2010) found that the increase of mathematics test scores for the ELL students corresponded to English-reading proficiency in a non-linear manner. ELL students' English-reading proficiency predicted mathematics test scores, progress in the online mathematics tutorial, and mathematics self-concept.

### Implications for Teachers and Educators

The current study can be used to inform mathematics teachers and educators on how best to respond to the instructional needs of their ELL students. In this study, mathematics self-efficacy was a significant predictor in six of 10 DIF items. Therefore, it is necessary for mathematics teachers and educators to develop this psychological belief for all students. Mathematics self-efficacy could be increased by using the right instructional strategies such as helping students to set learning goals, providing timely and explicit feedback, encouraging students to study harder, and using high achieving students as models (Liu and Hairy, 2009).

As a result of the language barrier and potentially negative perceptions of their academic ability from others, ELLs need additional support from mathematics teachers to enhance mathematics self-efficacy (Briscoe, 2014). Since the major sources of self-efficacy include mastery experience, vicarious experience, social persuasion, and psychological responses, it is helpful for ELLs to build self-efficacy by providing more successful experiences with mathematics, modeling, and verbal affirmations (Bandura, 1997).

The finding in the current study aligned with previous studies that proved language proficiency is a determinant factor to influence mathematics achievement (Abedi et al., 2001; Abedi, 2002; Haag et al., 2013; Loughran, 2014). It is suggested mathematics instruction should not isolate the word level from the discourse level. Teachers should provide opportunities for the discourse practices of explaining meanings of mathematical concepts and operations (Setati, 2005). The lexical support of meaning-related vocabulary offered in structured phrases rather than isolated words is important to the technical vocabulary (Prediger and Wessel, 2013).

### Implications for Test Developers

The current study also provided some implications for test developers in terms of assessment development. Testing organizations should be more aware of linguistic diversity within the student population to make academic assessments more accessible for ELL students (Sireci and Faulkner-Bond, 2015). Moreover, quantitative and qualitative control procedures should be included to facilitate validity for subgroups of students. The quantitative process should include item analysis to evaluate statistical qualities such as item difficulty and discrimination. The qualitative process can incorporate the sensitivity review, which is an independent review of tests and items by experts trained to consider the unique characteristics of important subgroups (Sireci and Faulkner-Bond, 2015). ELL students can also be interviewed and asked to explain why the pilot items confused them (Ilich, 2013).

## Conclusions

Examinations of DIF among language groups are a practical concern due to the increasing language diversity and the prevalence of testing. This study revealed that eight common items are identified with DIF effects using MH procedure, Rasch model, and HGLM. These eight items are all in favor of non-ELLs. These findings provided evidence supporting the claim that language ability has a negative impact on the mathematics performance for ELLs (Abedi, 2002; Martiniello, 2009; Loughran, 2014). Five items, identified by the Rasch model, was found to be in favor of ELLs. These items may be related to ELLs' prior educational experiences in their native languages. Although students are classified into ELLs as a result of their lack of English language proficiency, they may have been able to transfer key skills needed for those five items from their native languages to English.

When identifying the achievement gap between ELLs and non-ELLs, it is imperative to note that there are many possible reasons for the score differences. For instance, ELLs are more likely from low SES groups and may not have an equal chance to learn the content knowledge of mathematics. The unequal opportunities to learn result in true test score differences (Abedi et al., 2001). Inclusions of covariates in HGLM can solve this issue (Kamata, 2001). Finally, three items show strong evidence of DIF between ELLs and non-ELLs, even after controlling for student (e.g., mathematics self-efficacy, language proficiency, SES) and school (e.g., school type, school educational resources) level variables. Among the three items, two items (Item 40 and 46) with large DIF effect sizes (above 1.5) were categorized into Class C. According to ETS guidelines, items from Class C should not be used unless they are judged to be essential to meet test specifications (Zwick, 2012). Thus, it is suggested that PISA test developers should examine the language demand for these two items. Modifications or replacements should be made to reduce the DIF against ELLs.

The decreasing number of items showing DIF effects in HGLM Model 2 revealed that mathematics self-efficacy, language proficiency, and SES are potential sources of DIF between ELLs and non-ELLs. In addition, the number of DIF items continued to decrease after controlling for both student and school-level variables. This finding implied that DIF effects between ELLs and non-ELLs can vary in different schools. School type and school educational resources were also potential sources of DIF effects.

Since it is difficult to estimate the amount of error in the data (e.g., missing data) from empirical studies, applying more than one DIF detection approach was suggested to increase the confidence in the results (Hidalgo and LÓPez-Pina, 2004). There is some disagreement among three DIF detection approaches. This disagreement mainly resulted from the different mechanism of DIF detection methods. For instance, MH procedure used raw scores to match students from different groups for DIF detection, but raw scores cannot represent students' true ability levels properly when tests have DIF items or the impact is large (Jin et al., 2018). Besides, the MH procedure failed to make any assumptions about the classical test theory decomposition of scores. By comparison, Rasch model and HGLM can be classified into the parametric and the latent matching category. They are closely linked to a test theory that decomposes an observed score into a systematic true score and a stochastic error score (Kim, 2003). In terms of the Rasch model and HGLM, although the Rasch model has been regarded as a special case of HGLM (Kamata, 2001; Raudenbush et al., 2003), their detection mechanism of DIF effects is different. A general approach for detecting the DIF using the Rasch model is the Rasch separate calibration *t*-test method while HGLM relies on evaluating the interaction effect of item by person characteristic variable. Additionally, Kamata (2001) suggested that HGLM can be carried out by using one of the items as the reference item. As a result, within HGLM analyses, results may slightly vary due to the selection of the reference item.

For practitioners, it is important to note the roles of statistical DIF and substantive DIF (Peabody and Wind, 2019). All of the existing DIF detection methods are designed to identify statistical DIF. The analyst is expected to identify as many as possible to ensure that all items exhibit real DIF. After all items with the statistical DIF are identified, content experts will determine if substantive DIF exists.

## Limitations and Future Research

In this study, students whose primary language spoken at home was not English were classified as ELL students. While reading literacy was used to represent language proficiency and was controlled as a covariate in HGLM, levels of English proficiency for ELL students were unknown. Some students who were categorized into the ELL group may have transitioned out of English as a second language class. This variability within the ELL group may limit the results. It is recommended that large-scale assessments (e.g., PISA) can collect samples of data from students who are at varying levels of English language proficiency.

Potential sources of DIF between ELLs and non-ELLs should be interpreted with cautions. This study relied on correlational analysis to ascertain the relationship between DIF effects and other covariates. Further casual explanation for the achievement gap between ELLs and non-ELLs needs to be investigated at both student and school levels.

PISA only released a small portion of mathematics items so that it is impossible to review the detailed content of each item. Further comprehensive content analysis on the DIF items should be conducted when the PISA 2012 mathematics assessment items are released in the future. First, vocabulary and terminologies of DIF items should be reviewed by mathematics educators and assessment experts to see whether cultural bias exists. Second, content reviews can be made to rate the level of linguistic complexity by experts in the areas of literacy, linguistics, and bilingual education. Whether linguistic complexity can predict the magnitude of DIF effects between ELL and non-ELL students can be investigated.

## Data Availability Statement

Publicly available datasets were analyzed in this study. This data can be found at: https://www.oecd.org/pisa/pisaproducts/pisa2012database-downloadabledata.htm.

## Ethics Statement

The data included in this study was derived from PISA. Therefore, the study did not require ethics approval.

## Author Contributions

RL: generation of research goals and aims, development of research methodology, data analysis, and writing the draft. KB: providing mentorship on the research, reviewing, and editing the draft. All authors contributed to the article and approved the submitted version.

## Conflict of Interest

The authors declare that the research was conducted in the absence of any commercial or financial relationships that could be construed as a potential conflict of interest.

## Publisher's Note

All claims expressed in this article are solely those of the authors and do not necessarily represent those of their affiliated organizations, or those of the publisher, the editors and the reviewers. Any product that may be evaluated in this article, or claim that may be made by its manufacturer, is not guaranteed or endorsed by the publisher.
