# Supplementary material for: Differential Item Functioning Among English Language Learners on a Large-Scale Mathematics Assessment
Source: Front Psychol. 2021 Jul 27;12:657335. doi: 10.3389/fpsyg.2021.657335 (PMC8353367; doi:10.3389/fpsyg.2021.657335)
Supplement: Supplementary file 1 [file Data_Sheet_1.PDF]

Appendix A

Descriptions of Mathematics Assessment Items in PISA 2012

Item Number	PISA Variable Name	Description
1	PM00FQ01	MATH - P2012 Apartment Purchase Q1
2	PM00GQ01	MATH - P2012 An Advertising Column Q1
3	PM00KQ02	MATH - P2012 Wheelchair Basketball Q2
4	PM033Q01	MATH - P2000 A View with a Room Q1
5	PM034Q01T	MATH - P2000 Bricks Q1
6	PM155Q01	MATH - P2000 Pop Pyramids Q1
7	PM155Q04T	MATH - P2000 Pop Pyramids Q4
8	PM192Q01T	MATH - P2000 Containers Q1
9	PM273Q01T	MATH - P2000 Pipelines Q1
10	PM305Q01	MATH - P2000 Map Q1
11	PM406Q01	MATH - P2003 Running Tracks Q1
12	PM406Q02	MATH - P2003 Running Tracks Q2
13	PM408Q01T	MATH - P2003 Lotteries Q1
14	PM411Q01	MATH - P2003 Diving Q1
15	PM411Q02	MATH - P2003 Diving Q2
16	PM420Q01T	MATH - P2003 Transport Q1
17	PM423Q01	MATH - P2003 Tossing Coins Q1
18	PM442Q02	MATH - P2003 Braille Q2
19	PM446Q01	MATH - P2003 The Thermometer Cricket Q1
20	PM446Q02	MATH - P2003 The Thermometer Cricket Q2
21	PM462Q01D	MATH - P2003 The Third Side Q1
22	PM464Q01T	MATH - P2003 The Fence Q1
23	PM474Q01	MATH - P2003 Running Time Q1
24	PM496Q01T	MATH - P2003 Cash Withdrawal Q1
25	PM496Q02	MATH - P2003 Cash Withdrawal Q2
26	PM559Q01	MATH - P2003 Telephone Rates Q1
27	PM564Q01	MATH - P2003 Chair Lift Q1
28	PM564Q02	MATH - P2003 Chair Lift Q2
29	PM571Q01	MATH - P2003 Stop the Car Q1
30	PM603Q01T	MATH - P2003 Number Check Q1
31	PM800Q01	MATH - P2003 Computer Game Q1
32	PM803Q01T	MATH - P2003 Labels Q1
33	PM828Q01	MATH - P2003 Carbon Dioxide Q1
34	PM828Q02	MATH - P2003 Carbon Dioxide Q2
35	PM828Q03	MATH - P2003 Carbon Dioxide Q3
36	PM903Q03	MATH - P2012 Drip Rate Q3
37	PM905Q01T	MATH - P2012 Tennis Balls Q1
38	PM905Q02	MATH - P2012 Tennis Balls Q2

39	PM906Q01	MATH - P2012 Crazy Ants Q1
40	PM909Q01	MATH - P2012 Speeding Fines Q1
41	PM909Q02	MATH - P2012 Speeding Fines Q2
42	PM909Q03	MATH - P2012 Speeding Fines Q3
43	PM915Q01	MATH - P2012 Carbon Tax Q1
44	PM915Q02	MATH - P2012 Carbon Tax Q2
45	PM918Q01	MATH - P2012 Charts Q1
46	PM918Q02	MATH - P2012 Charts Q2
47	PM918Q05	MATH - P2012 Charts Q5
48	PM919Q01	MATH - P2012 Zs Fan Merchandise Q1
49	PM919Q02	MATH - P2012 Zs Fan Merchandise Q2
50	PM923Q01	MATH - P2012 Sailing Ships Q1
51	PM923Q03	MATH - P2012 Sailing Ships Q3
52	PM923Q04	MATH - P2012 Sailing Ships Q4
53	PM924Q02	MATH - P2012 Sauce Q2
54	PM943Q01	MATH - P2012 Arches Q1
55	PM943Q02	MATH - P2012 Arches Q2
56	PM949Q01T	MATH - P2012 Roof Truss Design Q1
57	PM949Q02T	MATH - P2012 Roof Truss Design Q2
58	PM953Q02	MATH - P2012 Flu Test Q2
59	PM953Q03	MATH - P2012 Flu Test Q3
60	PM954Q01	MATH - P2012 Medicine Doses Q1
61	PM954Q02	MATH - P2012 Medicine Doses Q2
62	PM954Q04	MATH - P2012 Medicine Doses Q4
63	PM955Q01	MATH - P2012 Migration Q1
64	PM955Q02	MATH - P2012 Migration Q2
65	PM982Q01	MATH - P2012 Employment Data Q1
66	PM982Q02	MATH - P2012 Employment Data Q2
67	PM982Q03T	MATH - P2012 Employment Data Q3
68	PM982Q04	MATH - P2012 Employment Data Q4
69	PM992Q01	MATH - P2012 Spacers Q1
70	PM992Q02	MATH - P2012 Spacers Q2
71	PM992Q03	MATH - P2012 Spacers Q3
72	PM995Q01	MATH - P2012 Revolving Door Q1
73	PM995Q02	MATH - P2012 Revolving Door Q2
74	PM995Q03	MATH - P2012 Revolving Door Q3
75	PM998Q02	MATH - P2012 Bike Rental Q2
76	PM998Q04T	MATH - P2012 Bike Rental Q4

Appendix B

Items of Mathematics Self-Efficacy

Question	Items
How confident do you feel about having to do the following tasks?	1. Using a train timetable to work out how long it would take to get from one place to another<br>2. Calculating how much cheaper a TV would be after a 30% discount<br>3. Calculating how many square meters of tiles you need to cover a floor<br>4. Understanding graphs presented in newspapers<br>5. Solving an equation like 3x+5= 17<br>6. Finding the actual distance between two places on a map<br>7. Solving an equation like 2(x+3) = (x + 3) (x - 3)<br>8. Calculating the petrol consumption rate of a car

Appendix C

Items of School Educational Resource Assessment

Question	Items
Is your school's capacity to provide instruction hindered by any of the following?	A lack of qualified science teachers
	A lack of qualified mathematics teachers
	A lack of qualified (test language) teachers
	A lack of teachers of other subjects
	A lack of laboratory technicians
	A lack of other support personnel
	Shortage or inadequacy of science laboratory equipment
	Shortage or inadequacy of instructional materials
	Shortage or inadequacy of computers for instruction
	Lack or inadequacy of Internet connectivity
	Shortage or inadequacy of computer software for instruction
	Shortage or inadequacy of library materials
	Shortage or inadequacy of audio-visual resources

Appendix D

Summary of Results from MH to Identify DIF Effects

Item Number	PISA Variable Name	MH Chi-Square	Odds-Ratio	DIF Effect Size	class
1	PM00FQ01	1.36	.84	.41	A
2	PM00GQ01	3.78	.47	1.78	A
3	PM00KQ02	.01	.97	.07	A
4	PM033Q01	.01	.98	.05	A
5	PM034Q01T	3.52	.72	.76	A
6	PM155Q01	5.03 *	.69	.87	A
7	PM155Q04T	8.16 *	.64	1.06	A
8	PM192Q01T	12.76 **	.55	1.42	B
9	PM273Q01T	2.86	.78	.60	A
10	PM305Q01	1.31	.84	.42	A
11	PM406Q01	1.75	.75	.67	A
12	PM406Q02	.50	.82	.46	A
13	PM408Q01T	2.23	.79	.57	A
14	PM411Q01	7.40 *	.66	.98	A
15	PM411Q02	7.60 *	.68	.89	A
16	PM420Q01T	21.23 **	.50	1.63	C
17	PM423Q01	2.41	.77	.63	A
18	PM442Q02	2.88	.75	.68	A
19	PM446Q01	4.47 *	.68	.89	A
20	PM446Q02	2.21	.57	1.31	A
21	PM462Q01D	1.63	.82	.47	A
22	PM464Q01T	.92	.81	.50	A
23	PM474Q01	.01	.98	.05	A
24	PM496Q01T	6.68 *	.67	.94	A
25	PM496Q02	3.92 *	.73	.73	A
26	PM559Q01	3.96 *	.74	.71	A
27	PM564Q01	.06	1.04	-.09	A
28	PM564Q02	2.41	.78	.59	A
29	PM571Q01	2.05	.80	.53	A
30	PM603Q01T	5.07 *	.69	.86	A
31	PM800Q01	3.80	.71	.82	A
32	PM803Q01T	3.13	.72	.78	A
33	PM828Q01	6.57 *	.68	.92	A
34	PM828Q02	.09	1.05	-.11	A
35	PM828Q03	1.31	.81	.51	A
36	PM903Q03	2.54	.77	.61	A
37	PM905Q01T	7.68 *	.64	1.06	A
38	PM905Q02	5.23 *	.68	.91	A

39	PM906Q01	.32	.91	.21	A
40	PM909Q01	14.84 **	.39	2.24	C
41	PM909Q02	20.02 **	.51	1.59	C
42	PM909Q03	31.67 **	.34	2.57	C
43	PM915Q01	13.10 **	.55	1.42	B
44	PM915Q02	.02	1.02	-.05	A
45	PM918Q01	5.51 *	.67	1.33	A
46	PM918Q02	23.31 **	.47	1.80	C
47	PM918Q05	7.78 **	.64	1.06	B
48	PM919Q01	2.69	.76	.65	A
49	PM919Q02	10.10 **	.62	1.12	B
50	PM923Q01	2.25	.81	.51	A
51	PM923Q03	.39	1.09	-.21	A
52	PM923Q04	.65	.83	.44	A
53	PM924Q02	3.36	.77	.62	A
54	PM943Q01	.44	.91	.22	A
55	PM943Q02	1.50	1.63	-1.15	A
56	PM949Q01T	14.22 **	.56	1.35	B
57	PM949Q02T	14.00 **	.50	1.61	C
58	PM953Q02	6.04 *	.70	.83	A
59	PM953Q03	4.83 *	.73	.76	A
60	PM954Q01	.77	.87	.32	A
61	PM954Q02	16.74 **	.51	1.59	C
62	PM954Q04	5.21 *	.66	.96	A
63	PM955Q01	16.90 **	.51	1.57	C
64	PM955Q02	11.75 **	.48	1.73	C
65	PM982Q01	.00	1.01	-.02	A
66	PM982Q02	.02	1.03	-.06	A
67	PM982Q03T	.59	.88	.29	A
68	PM982Q04	9.52 **	.60	1.20	B
69	PM992Q01	2.32	.76	.65	A
70	PM992Q02	.55	.84	.41	A
71	PM992Q03	.14	.88	.31	A
72	PM995Q01	4.33 *	.74	.71	A
73	PM995Q02	5.11 **	.18	4.05	C
74	PM995Q03	8.17 **	.66	.99	A
75	PM998Q02	2.76	.74	.72	A
76	PM998Q04T	.10	1.05	-.12	A

Note: *p≤.05; ** p≤.01

Appendix E

Summary of Results from Rasch Model to Identify DIF Effects

Item Number	PISA Variable Name	Difficulty Measures		Difficulty Contrast	t
		Non-ELL	ELL		
1	PM00FQ01	.39	.26	.13	.49
2	PM00GQ01	3.36	3.51	-.15	.75
3	PM00KQ02	3.13	2.78	.35	.32
4	PM033Q01	-1.69	-2.05	.36	.07
5	PM034Q01T	.56	.56	.00	1.00
6	PM155Q01	-1.49	-1.42	-.07	.70
7	PM155Q04T	-.67	-.49	-.19	.31
8	PM192Q01T	.25	.66	-.40 *	.05
9	PM273Q01T	-.32	-.51	.19	.28
10	PM305Q01	-.18	-.37	.19	.30
11	PM406Q01	1.72	1.72	.00	1.00
12	PM406Q02	2.59	2.48	.11	.72
13	PM408Q01T	.58	.38	.20	.29
14	PM411Q01	-.25	-.08	-.16	.39
15	PM411Q02	-.32	-.15	-.17	.37
16	PM420Q01T	-.88	-.48	-.40 *	.02
17	PM423Q01	-1.85	-1.93	.08	.67
18	PM442Q02	.50	.47	.03	.87
19	PM446Q01	-1.82	-1.68	-.14	.44
20	PM446Q02	3.33	3.61	-.27	.53
21	PM462Q01D	-1.16	-1.42	.25	.15
22	PM464Q01T	2.04	1.89	.15	.57
23	PM474Q01	-1.45	-1.82	.37	.06
24	PM496Q01T	-.47	-.37	-.09	.60
25	PM496Q02	-1.05	-1.07	.02	.90
26	PM559Q01	-.74	-.85	.11	.53
27	PM564Q01	.09	-.39	.48 *	.01
28	PM564Q02	.13	.06	.07	.71
29	PM571Q01	-.14	-.24	.10	.58
30	PM603Q01T	.12	.19	-.07	.72
31	PM800Q01	-2.11	-2.14	.03	.88
32	PM803Q01T	1.09	1.09	.00	1.00
33	PM828Q01	.81	.89	-.08	.72
34	PM828Q02	-.79	-1.37	.58 **	.00
35	PM828Q03	1.41	1.22	.19	.38
36	PM903Q03	.91	.91	.00	1.00
37	PM905Q01T	-2.18	-1.91	-.27	.14
38	PM905Q02	.26	.44	-.18	.33

39	PM906Q01	-.33	-.50	.17	.35
40	PM909Q01	-3.62	-3.08	-.54 *	.03
41	PM909Q02	-.72	-.50	-.23	.20
42	PM909Q03	.82	1.56	-.74 **	.00
43	PM915Q01	-.06	.42	-.48 *	.01
44	PM915Q02	-.44	-.76	.32	.08
45	PM918Q01	-3.46	-3.29	-.17	.49
46	PM918Q02	-2.04	-1.50	-.53 **	.00
47	PM918Q05	-1.90	-1.80	-.10	.60
48	PM919Q01	-2.19	-2.16	-.03	.87
49	PM919Q02	.19	.49	-.30	.09
50	PM923Q01	-.30	-.41	.11	.54
51	PM923Q03	.14	-.41	.54 *	.00
52	PM923Q04	2.48	2.42	.06	.83
53	PM924Q02	-.36	-.40	.04	.81
54	PM943Q01	-.41	-.63	.22	.19
55	PM943Q02	4.69	3.66	1.03 **	.02
56	PM949Q01T	-.82	-.75	-.07	.68
57	PM949Q02T	.91	1.12	-.21	.35
58	PM953Q02	-.28	-.18	-.10	.55
59	PM953Q03	-.03	.03	-.05	.75
60	PM954Q01	-1.55	-1.66	.11	.53
61	PM954Q02	.65	1.21	-.56 **	.01
62	PM954Q04	1.31	1.48	-.17	.42
63	PM955Q01	-1.74	-1.52	-.22	.22
64	PM955Q02	1.54	1.80	-.26	.33
65	PM982Q01	-2.79	-3.01	.23	.39
66	PM982Q02	.72	.34	.39 *	.05
67	PM982Q03T	-.89	-.99	.10	.60
68	PM982Q04	.01	.36	-.35	.07
69	PM992Q01	-1.87	-1.77	-.10	.61
70	PM992Q02	2.07	1.91	.16	.55
71	PM992Q03	3.41	3.17	.24	.55
72	PM995Q01	-.13	-.13	.00	1.00
73	PM995Q02	4.58	5.96	-1.38	.08
74	PM995Q03	.01	.21	-.19	.28
75	PM998Q02	-2.16	-2.38	.22	.27
76	PM998Q04T	.81	.03	.78 **	.00

Note: *p≤.05; **p≤.01
